# Astrocyte-Derived Proinflammatory Cytokines Induce Hypomyelination in the Periventricular White Matter in the Hypoxic Neonatal Brain

**DOI:** 10.1371/journal.pone.0087420

**Published:** 2014-01-31

**Authors:** Yiyu Deng, Di Xie, Ming Fang, Gaofeng Zhu, Chunbo Chen, Hongke Zeng, Jia Lu, Kaur Charanjit

**Affiliations:** 1 Department of Critical Care and Emergency, Guangdong General Hospital, Guangdong Academy of Medical Sciences, Guangzhou, PR China; 2 Graduate School, Southern Medical University, Guangzhou, PR China; 3 Defense Medical and Environmental Research Institute, DSO National Laboratories, Singapore; 4 Department of Anatomy, Yong Loo Lin School of Medicine, National University of Singapore, Singapore; Univ. Kentucky, United States of America

## Abstract

Hypoxic exposure in the perinatal period causes periventricular white matter damage (PWMD), a condition associated with myelination abnormalities. Under hypoxic conditions, glial cells were activated and released a large number of inflammatory mediators in the PWM in neonatal brain, which may result in oligodendrocyte (OL) loss and axonal injury. This study aims to determine if astrocytes are activated and generate proinflammatory cytokines that may be coupled with the oligodendroglial loss and hypomyelination observed in hypoxic PWMD. Twenty-four 1-day-old Wistar rats were exposed to hypoxia for 2 h. The rats were then allowed to recover under normoxic conditions for 7 or 28 days before being killed. Another group of 24 rats kept outside the chamber was used as age-matched controls. Upregulated expression of TNF-α and IL-1β was observed in astrocytes in the PWM of P7 hypoxic rats by double immunofluorescence, western blotting and real time RT-PCR. This was linked to apoptosis and enhanced expression of TNF-R_1_ and IL-1R_1_ in APC^+^ OLs. PLP expression was decreased significantly in the PWM of P28d hypoxic rats. The proportion of myelinated axons was markedly reduced by electron microscopy (EM) and the average g-ratios were higher in P28d hypoxic rats. Upregulated expression of TNF-α and IL-1β in primary cultured astrocytes as well as their corresponding receptors in primary culture APC^+^ oligodendrocytes were detected under hypoxic conditions. Our results suggest that following a hypoxic insult, astrocytes in the PWM of neonatal rats produce inflammatory cytokines such as TNF-α and IL-1β, which induce apoptosis of OLs via their corresponding receptors associated with them. This results in hypomyelination in the PWM of hypoxic rats.

## Introduction

Periventricular white matter damage (PWMD) is the major neuropathological form of brain injury observed in preterm infants, and is the leading cause of cerebral palsy [1]–[3]. The pathogenesis of PWMD is complex and not fully understood at present, but hypoxia-ischemia and infection have been known as two primary factors [4]–[6]. We previously reported that PWMD caused by hypoxia has been associated with microglial activation, diffuse reactive astrogliosis, axonal injury, oligodendrocyte (OL) progenitor apoptosis and myelination disturbances [7]–[9]. Inflammation triggered by hypoxia plays a crucial role in the pathogenesis of PWMD [7]–[9]. Our previous studies have also shown that a strong and persistent inflammation occurred in the periventricular white matter (PWM) in hypoxic neonatal brain, which is closely associated with axonal disruption and pre-OLs injury [10], [11].

The glial cells in hypoxic neonatal brain were activated and released excessive inflammatory mediators, which disturbed the physiological process of myelination by attacking the axons and pre-oligodendrocytes (pre-OLs) [12], [13]. It has been well documented that activated microglial cells and astrocytes may be main source of inflammatory mediators in neonatal brain under pathological conditions [14], [15]. Amoeboid microglial cells (AMCs) are resident immune cells in the central nervous system (CNS) [16], [17]. Previous studies have demonstrated that AMCs were activated and released a large number of inflammatory cytokines and chemokines in the PWM at P1-7d after hypoxic exposure [10], [11], [16]. The number of activated microglial cells was markedly increased by migration of cells from blood or other brain regions, which would augment the inflammatory response occurring in the PWM of the hypoxic neonatal rats [16]. Astrocytes are closely associated with vascular development, formation of synapses and blood-brain barrier (BBB) within specific regions including PWM under physiological conditions [18]–[20]. Following different kinds of injury or in neurodegenerative diseases, astrocytes may produce reactive responses [21]. It has been reported that astrocytic reactivity occurs within the deep white matter in the hypoxic developing brain, which is coupled with widespread axonal damage and production of the pro-inflammatory cytokines [2], [21]. Astrocytic responses have the potential to overproduce free radicals and inflammatory mediators such as TNF-α and IL-1β [2]. However, the role of astrocytes in PWMD in neonatal brain under hypoxic conditions has not been fully addressed. This study aims to explore if astrocytes are activated and contribute to the occurrence of hypomyelination under hypoxic conditions in the neonatal brain. Expression of cytokines such as TNF-α and IL-1β in astrocytes was first observed by double immunofluorescence. We then ascertained whether the above-mentioned proinflammatroy cytokines would induce the apoptosis of OLs through their corresponding receptors such as TNF-R_1_ and IL-1R_1_ expressed in OLs. Myelin proteolipid protein (PLP) expression was observed in the PWM of P28d control and hypoxic rats. The proportion of myelinated axons and thickness of myelin sheath was examined in hypoxic rats in comparison with the controls by electron microscopy. We report here that astrocytes are one of the main sources of proinflammatory cytokines that may contribute to inducing hypomyelination, a salient characteristic feature of PWMD.

## Materials and Methods

### Animals

One-day old Wistar rats were used in this study because it has been reported that the developmental stage of white matter in rats at birth is equivalent to mid-gestation in humans [22]. Twenty four 1-day old Wistar rats were subjected to hypoxia by placing them in a chamber filled with a gas mixture of 5% oxygen and the remainder 95% nitrogen (Model: MCO 18 M; Sanyo® Biomedical Electrical Co., Ltd, Tokyo, Japan.) for 2 h. The rats were then allowed to recover under normoxic conditions for 7 or 28 days before being killed. Another group of 24 rats kept outside the chamber was used as age-matched controls. The rats in each group were perfused and fixed. The brains were removed from the rats and 20 um sections were cut for double immunofluorescence. Animal handling and experiments were approved by Institutional Animal Care and Use Committee, Guangdong Province, China.

### Mixed Glial Cell Culture

Mixed glial cells separated from the cerebral cortex of 1-day old postnatal Wistar rats were plated on a 75 cm^2^ flask at a density of 1.2×10^6^ cells/mL of Dulbecco’s Modified Eagle’s Medium (DMEM, Sigma, St. Louis, MO, USA) supplemented with 10% fetal bovine serum (FBS, Hyclone®, Logan, UT, USA). The culture medium was changed after 24 h and then twice a week.

### Astrocyte Purification and Treatment

Two weeks later, nonastroglial cells were removed by shaking [23]. For immunocytochemistry, purified astrocytes in the 75 cm^2^ flask were isolated by trypsinization, and the detached cells were plated at 2.5×10^5^ per well on a 24 multi-well culture dish and incubated at 37°C in a humidified atmosphere of 95% air and 5% CO_2_ for 24 h. For western blotting and mRNA extraction, purified cells were plated at 1×10^6^ cells per flask. On the following day, the cells were subjected to different treatments. The purity of astrocyte culture was determined by double labeling of cultured cells with glial fibrillary acidic protein (GFAP) (1∶1000: Chemicon® International Temecula, CA, USA), a widely used marker for rat astrocytes, and DAPI (20 µg/mL; Sigma), a nuclear marker of all cells. This immunocytochemical analysis indicated that more than 99% of the confluent cells were GFAP-positive astrocytes. After treatment, astrocytes were used to study the effects of hypoxia on expression of TNF-α and IL-1β in the astrocytes. For this, the cells were exposed in a hypoxia chamber (model MCO18 Multi-gas incubator, Sanyo Company Pte. Ltd., Tokyo, Japan) for 3 h at 3% oxygen, 5% CO_2_ and 92% nitrogen at 37°C.

### Oligodendrocyte Purification and Treatment

Ten days after culture, oligodendrocytes were purified by shaking [23]. For immunocytochemistry, purified oligodendrocytes in the 75 cm^2^ flask were isolated, and the detached cells were plated at 2.5×10^5^ per well on a 24 multi-well culture dish and incubated at 37°C in a humidified atmosphere of 95% air and 5% CO_2_ for 24 h. After treatment, oligodendrocytes were used to study the effects of hypoxia on expression of TNF-R1 and IL-1R1 in the oligodendrocytes. For this, the cells were exposed in a hypoxia chamber (model MCO18 Multi-gas incubator, Sanyo Company Pte. Ltd., Tokyo, Japan) for 3 h at 3% oxygen, 5% CO_2_ and 92% nitrogen at 37°C.

### Real-time RT-PCR

Total RNA was extracted from PWM of the hypoxic rats (n = 5 at 7 days) and their age-matched controls (n = 5 at 7 days) or from cultured astrocytes using RNAesy mini kit® (Qiagen, Valencia, CA, USA) according to the manufacturer’s protocol. The quantity of total RNA was measured with a Biophotometer® (Eppendorf, CA, USA). For the reverse transcription, 2 µg of total RNA was combined with 1 µm of Oligo (dT) 15 primer (Invitrogen, Carlsbad, CA, USA) mixture then heated at 70°C for 5 minutes and then placed on ice. Single-strand cDNA was synthesized from the RNA by adding the following reagents (final concentrations): 1× first strand buffer, 1 U/µL RNAsin®, 25 µm of each Deoxyribonucleotide triphosphate (dNTP) and 200 U M-MLV reverse transcriptase® (Promega, Madison, WI, USA). The reaction mixture (20 µL) was incubated at 42°C for 50 minutes and the mixture was heated to 95°C for 5 minutes in order to terminate the reaction. The samples were stored at −20°C for PCR analysis.

Quantitative RT-PCR was carried out on a LightCycler 3® instrument using a FastStart DNA Master plus SYBR Green I kit® (Roche Diagnostics, GmbH, Roche Applied Science, Mannheim, Germany) according to the manufacturer’s instructions. Briefly, a LightCycler 3 master mix was prepared using 2 µL Light Cycler-FastStart DNA Master plus SYBR Green I with forward and reverse primers at a final concentration of 0.5 µM each. A 2-µL sample of the cDNA was added into 18 µL of LightCycler master mix and transferred into LightCycler glass capillaries. The capillaries were capped, placed in a LightCycler carousel and centrifuged in a specific LightCycler centrifuge. Thermal cycling was carried out. The first segment of the amplification cycle consisted of a denaturation program at 95°C for 10 minutes. The second segment consisted of denaturation, primer annealing, elongation and a quantification program repeated for 35 cycles. The third segment consisted of a melting-curve program. The final segment consisted of a cooling program at 40°C.

The expression of target genes was measured in triplicate and was normalized to β-actin as an internal control. Forward and reverse primer sequences for each gene and their corresponding amplicon size are provided in Table 1. Gene expression was quantified using a modification of the 2^−ΔΔct^ method as previously described [24].

**Table 1:** Sequence of specific primers.

Primer	Forward	Reverse	Ampliconsize
TNF-α	ccaacaaggaggagaagttcc	ctctgcttggtggtttgctac	134 bp
IL-1β	ggaacccgtgtcttcctaaag	ctctgcttggtggtttgctac	123 bp
TNF-R_1_	ggcccacttctaatgtgtgaa	aggctacaagagggagacagc	163 bp
IL-1R_1_	taaacctctgcctcttgacga	tgcttcccctggtatgtgtag	146 bp
β-actin	tcatgaagtgtgacgttgacatccgt	cctagaagcatttgcggtgcaggatg	285 bp

Abbreviations: TNF-α = tumor necrosis factor-α; IL-1β = interleukin-1β; TNF-R_1_ = TNF receptor 1; IL-1R_1_ = IL-1 receptor 1.

### Western Blotting

Proteins were extracted from PWM of the hypoxic rats (n = 5 at 7 days, n = 5 at 28 days) and their age-matched controls (n = 5 at 7 days, n = 5 at 28 days) or from astrocytes using a protein extraction kit (Pierce Biotechnology Inc, IL, USA) according to the manufacturer’s protocol. Protein concentrations were determined by the Bradford method using bovine serum albumin (BSA) (Sigma-Aldrich, St Louis, MO, USA) as a standard. Samples of supernatants containing 20 µg of protein were heated to 95°C for 5 minutes and were separated by sodium dodecyl sulfate–polyacrylamide gel electrophoresis in 12% gels in a Mini-Protein 3 apparatus (Bio-Rad Laboratories, Hercules, CA, USA). Protein bands were electroblotted onto 0.45-µm polyvinylindene difluoride membranes (Bio-Rad) at 1.5 mA/cm^2^ of membrane for 1 h in Towbin buffer, pH 8.3, to which 20% (v/v) methanol had been added. After transfer, the membranes were blocked with 5% (mass/vol) nonfat dried milk in Tris-buffered saline containing 0.05% Tween 20 (TBST) [0.05% (v/v) Tween-20 in 20 mm Tris-HCl buffer, pH 7.6, containing 137 mm sodium chloride] for 1 h, then incubated with the primary antibodies according to the manufacturer’s recommendations. The primary antibodies used were as follows: TNF-α (rabbit polyclonal IgG 1∶1000) (Chemicon International, Temecula, CA, USA; Cat. No.AB1837P), IL-1β (rabbit polyclonal IgG 1∶5000) (Chemicon International; Cat. No. AB1832P), TNF-R_1_ (rabbit polyclonal IgG 1∶500) (Santa Cruz Biotechnology, Santa Cruz, CA, USA; Cat. No.sc-7895), IL-1R_1_ (rabbit polyclonal IgG 1∶500) (Santa Cruz Biotechnology; Cat. No.sc-689), PLP (goat polyclonal IgG 1∶200) (Santa Cruz, CA, USA; Cat. No. sc-23570) and β-actin (mouse monoclonal IgG 1∶5000) (Sigma; Cat. No. A2172). After three washes with Tris-buffered saline with 0.1% Tween-20, the membranes were incubated with the horseradish peroxidase (HRP)-conjugated secondary antibodies (Cell Signaling Technology; Cat. No 7074) for 1 h. The immunoblots were developed using the enhanced chemiluminescence detection system (Pierce Biotechnology Inc., Rockford, IL, USA). Blots were stripped with stripping buffer (Pierce Biotechnology Inc.; Cat. No. 0021059) and hybridized with total kinases or β-actin. The signal intensity of TNF-α, IL-1β, TNF-R_1_, IL-1R_1_ and PLP levels relative to control were measured with Quantity One Software®, version 4.4.1 (BioRad Laboratories).

### Double Immunofluorescence

The sections from P7d control and P7d hypoxic rats (n = 3 in each group at 7 days) were divided into three groups. The sections in group I were then incubated with antibodies directed against anti-TNF-α, (rabbit polyclonal IgG 1∶1000) (Chemicon International, Temecula, CA, USA; Cat. No. AB1837P), anti-IL-1β (rabbit polyclonal IgG 1∶5000) (Chemicon International; Cat. No. AB1832P) and GFAP (mouse polyclonal IgG 1∶1000) (Chemicon International, Temecula, CA, USA; Cat. No. MAB360). The sections in group II were incubated with a mix of anti-TNF-R_1_ (rabbit polyclonal IgG 1∶500) (Santa Cruz Biotechnology, Santa Cruz, CA, USA; Cat. No.sc-7895), anti-IL-1R_1_ (rabbit polyclonal IgG 1∶500) (Santa Cruz Biotechnology; Cat. No.sc-689) and APC monoclonal antibody against adenomatus polyposis coli (APC) (oncogene, mouse monoclonal IgG 1∶20; Cat.No.OP80). The sections in group III were incubated with a mix of cleaved caspase-3 (Abcam, rabbit monoclonal IgG 1∶100; Cat.No. ab32351) and APC monoclonal antibody against adenomatus polyposis coli (APC) (oncogene, mouse monoclonal IgG 1∶20; Cat.No.OP80). Cleaved caspase-3 staining specifically stains the apoptotic cells. All incubations were at room temperature. On the following day, the sections were washed and incubated with a secondary antibody: CY3-conjugated goat anti-rabbit IgG (1∶100; Sigma, Cat. No. AP307 F) or CY3-conjugated rabbit anti-mouse IgG (1∶100, Chemicon, International; Cat. No. AP106C) for 1 h. After this, the sections were washed and incubated with fluorescein isothiocyanate (FITC)-conjugated goat anti-mouse (1∶100 Chemicon International; Cat. No. AP130F) or FITC-conjugated donkey anti-rabbit (1∶100 Santa Cruz Biotechnology; Cat. No.sc-2090) for 1 h. The sections from P28d control and P28d hypoxic rats (n = 3 in each group at 28 days) were incubated with PLP (goat polyclonal IgG 1∶200) (Santa Cruz, CA, USA; Cat. No. sc-23570) overnight at room temperature. On the following day, the sections were washed and incubated with a secondary antibody: CY3-conjugated rabbit anti-goat IgG (1∶100) for 1 h at 37°C in the dark. Finally, these sections were mounted with a fluorescent mounting medium (DAKO Cytomation, Glostrup, Denmark). Cellular colocalization was examined by confocal microscopy (LSM FV1000; Olympus® Company Pte Ltd., Tokyo, Japan).

The number of APC and caspase-3 positive oligodendrocytes in the corpus callosum was calculated by counting eight randomly selected microscopic fields in sections obtained from each rat (n = 3) at ×40 objective by a blinded observer. The percentage of caspase-3 positive oligodendrocytes against the total number of APC positive oligodendrocytes was calculated and averaged.

For cultured astrocytes, the cells were treated with 3% oxygen either for 3 h (for TNF-α or IL-1β detection). After the hypoxic treatment, the cells were fixed in 4% paraformaldehyde for 30 minutes, blocked in 1% BSA for 30 minutes and incubated with primary antibodies overnight at 4°C. Immunofluorescence labeling was carried out using primary antibodies directed against TNF-α (rabbit polyclonal IgG 1∶200; Cat. No. AB1837P), IL-1β (rabbit polyclonal IgG 1∶200; Cat. No.AB1832P) and GFAP (Chemicon International, mouse polyclonal IgG 1∶1000; Cat. No. MAB360). The cells were then incubated with CY3-conjugated goat-anti-rabbit secondary antibody (1∶100, Sigma; Cat. No. AP307 F) and with fluorescein isothiocyanate (FITC)-conjugated goat anti-mouse (1∶100 Chemicon International; Cat. No. AP130F) for 1 h. For cultured oligodendrocytes, the cells were treated with 3% oxygen either for 3 h (for TNF-R1 or IL-1R1 detection). After the hypoxic treatment, the cells were fixed in 4% paraformaldehyde for 30 minutes, blocked in 1% BSA for 30 minutes and incubated with primary antibodies overnight at 4°C. Immunofluorescence labeling was carried out using primary antibodies directed against TNF-R1 (rabbit polyclonal IgG 1∶500) (Santa Cruz Biotechnology, Santa Cruz, CA, USA; Cat. No.sc-7895), anti-IL-1R_1_ (rabbit polyclonal IgG 1∶500) (Santa Cruz Biotechnology; Cat. No.sc-689) and APC monoclonal antibody against adenomatus polyposis coli (APC) (oncogene, mouse monoclonal IgG 1∶20; Cat.No.OP80). The cells were then incubated with CY3-conjugated goat-anti-rabbit secondary antibody (1∶100, Sigma; Cat. No. AP307 F) and with fluorescein isothiocyanate (FITC)-conjugated goat anti-mouse (1∶100 Chemicon International; Cat. No. AP130F) for 1 h. Finally, the cells were counterstained with DAPI and examined under a confocal microscope.

### Electron Microscopy

The hypoxic (n = 3 at 28 days) and the corresponding control rats (n = 3 at 28 days) were perfused with a mixed aldehyde fixative composed of 2% paraformaldehyde and 3% glutaraldehyde in 0.1 M phosphate buffer, pH 7.2. After perfusion, the brain was removed and coronal slices (approximately 1 mm thick) were cut. Blocks of corpus callosum were trimmed from these slices. Vibratome sections (Model 3000™, The Vibratome Company, St Louis, MO, USA) of 80–100 µm thickness were prepared from these blocks and rinsed overnight in 0.1 M phosphate buffer. They were then postfixed for 2 h in 1% osmium tetraoxide, dehydrated and embedded in Araldite mixture. Ultrathin sections were cut and viewed in a Philips CM 120 electron microscope (FEI Company, Hillsboro, Oregon, USA).

Four nonoverlapping regions of the medial corpus callosum from each animal were photographed at different magnifications. The diameter of each axon fiber and axon fiber plus myelin sheath was measured by using Image J software (SummaSketch III Summagraphics, Seattle, WA). The g-ratio was calculated per axon as axon diameter to total fiber diameter (It is equivalent to axon area/axon+myelin sheath area) by a researcher blind to control/hypoxic group and compared between groups with a student’s t test (a = 0.05, two-tailed; n = 3 per group).

### Statistical Analysis

The data were presented as means ±SD. Statistical significance was evaluated by the Student’s t-test. Results were considered as significant at p<0.05.

## Results

### Western Blotting and Real Time RT-PCR Analysis of TNF-α, IL-1β, TNF-R_1_, IL-1R_1_ Protein and mRNA Expression in the PWM

**Figure 1:**
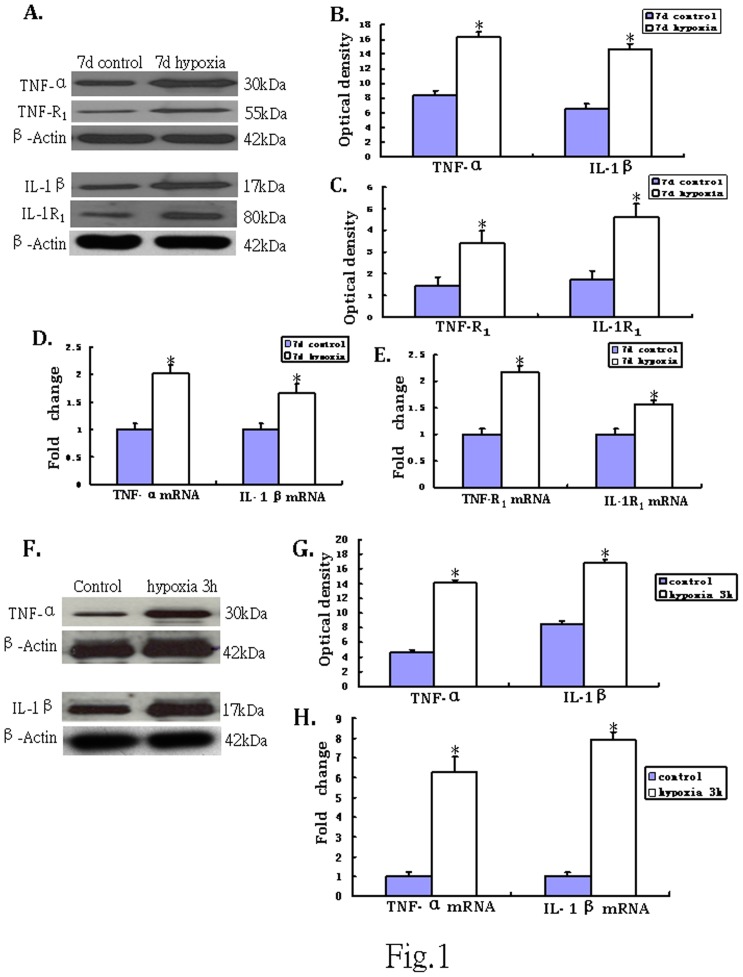
TNF-α and IL-1β, TNF-R_1_, IL-1R_1_ protein and mRNA expression in the PWM of P7d rats *in vivo* and in the astrocytes *in vitro*. A shows TNF-α (30 kDa) and TNF-R_1_ (55 kDa), IL-1β (17 kDa), IL-1R_1_ (80 kDa) and β-actin (42 kDa) immunoreactive bands, respectively. Bar graphs in B, C show significant increase in the optical density of TNF-α and IL-1β, TNF-R_1_, IL-1R_1_ following hypoxic exposure when compared with the corresponding controls (*P<0.01). Panels D and E show the graphical representation of the fold changes in TNF-α and IL-1β, TNF-R_1_, IL-1R_1_ mRNA, respectively as quantified by normalization to the β-actin as an internal control. Significant increase in TNF-α and IL-1β, TNF-R_1_, IL-1R_1_ mRNA levels in the PWM after the hypoxic exposure is evident when compared with controls (*P<0.01). The panels F show TNF-α (30 kDa) and IL-1β (17 kDa) immunoreactive protein bands in cultured control astrocytes and at 3 h after hypoxic exposure. G is bar graph showing changes in the optical density of TNF-α and IL-1β, respectively, following hypoxic exposure. H (TNF-α and IL-1β mRNA) show the graphical representation of the fold changes quantified by normalization to the β-actin as an internal control. Significant differences in protein and mRNA levels in astrocytes after the hypoxic exposure are evident when compared with controls (* *P*<0.01).

The immunoreactive bands of TNF-α and IL-1β protein levels that appeared at approximately 30 kDa and 17 kDa, respectively (Fig. 1.A), increased significantly (p<0.05) in optical density at 7 d after hypoxic exposure as compared with the controls (Fig. 1.B). The immunoreactive band of TNF-R_1_ protein levels appeared at approximately 55 kDa (Fig. 1A). The optical density was increased significantly (p<0.05) at 7d after hypoxic exposure as compared with the controls (Fig. 1C). The immunoreactive band of IL-1R_1_ protein levels appeared at approximately 80 kDa (Fig. 1A). It was augmented significantly (p<0.05) at 7d after hypoxic exposure when compared with the controls (Fig. 1C).

TNF-α mRNA expression was significantly increased in the corpus callosum at 7d after the hypoxic exposure in comparison with the corresponding controls (p<0.05) (Fig. 1D). Increase in IL-1β mRNA expression paralleled that of TNF-α mRNA after the hypoxic exposure at 7d as compared with the matching controls (p<0.05) (Fig. 1D). TNF-R_1_ mRNA expression was markedly increased at 7 days in hypoxic rats when compared with the controls (p<0.05) (Fig. 1 E.). Increase in IL-1R_1_ mRNA expression was also observed at 7d after the hypoxic exposure in comparison with the controls (p<0.05) (Fig. 1E).

### Cellular Localization of TNF-α, IL-1β, TNF-R_1_ and IL-1R_1_ Protein Expression in the PWM by Double Labeling

**Figure 2:**
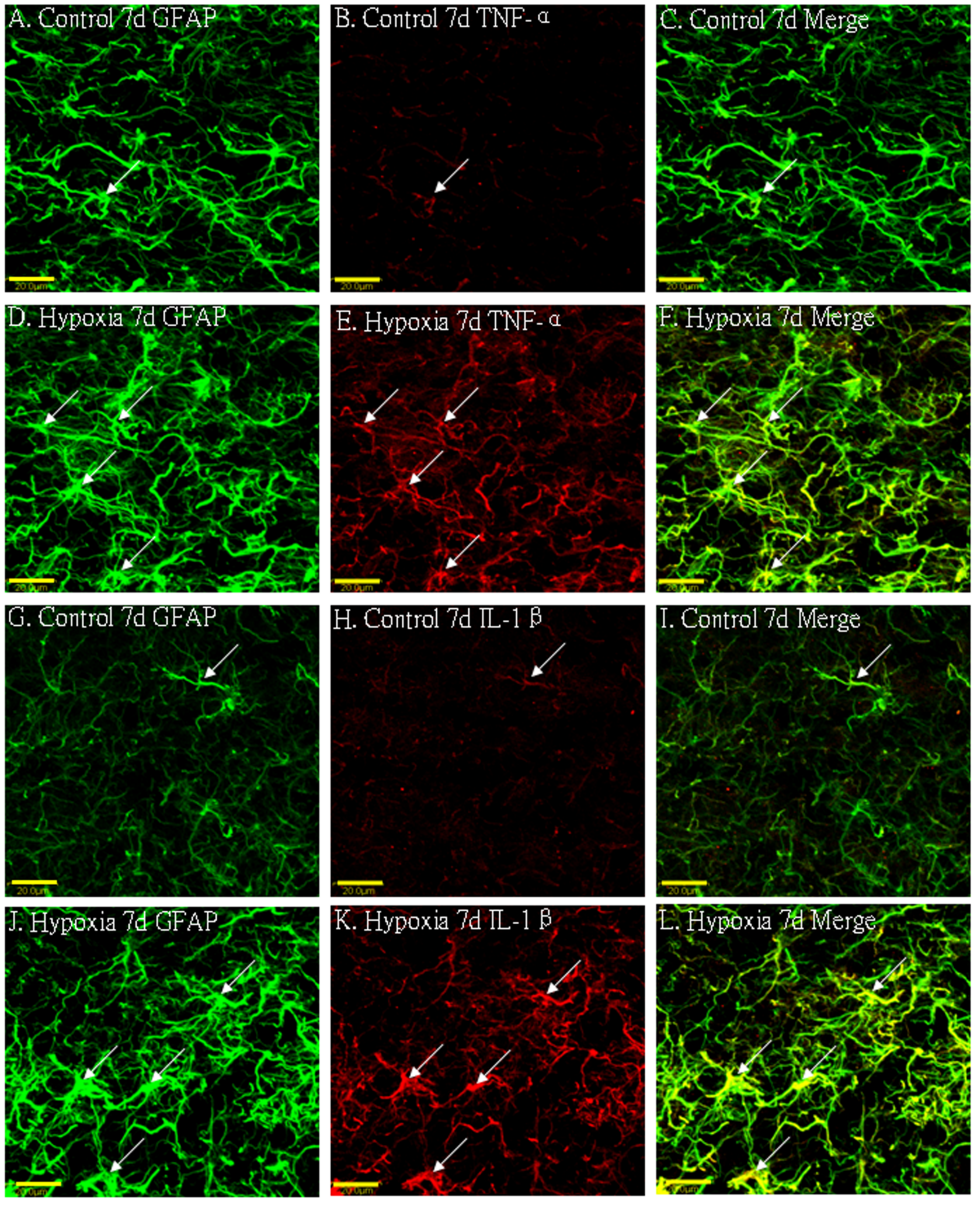
Confocal images showing the distribution of GFAP-labeled (A, D, G, J green), and TNF-α (B, E red), IL-1β (H, K red) immunoreactive astrocytes (arrows) in the PWM at 7 days after the hypoxic exposure and the corresponding control rats. The co-localized expression of GFAP and TNF-α, IL-1β in astrocytes can be seen in panels C, F, I and L. Note TNF-α and IL-1β expression in astrocytes (arrows) is markedly enhanced at 7 days after the hypoxic exposure. Scale bars: A-L, 20 µm. GFAP = glial fibrillary acidic protein; PWM = periventricular white matter.

**Figure 3:**
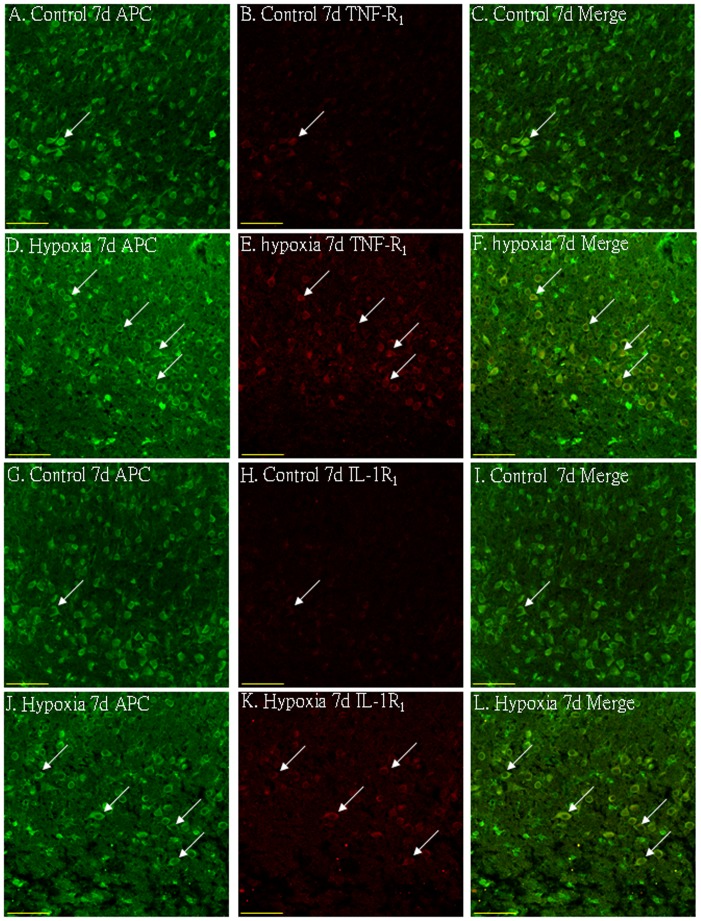
Confocal images showing the distribution of APC (A, D, G, J, green), tumor necrosis factor receptor 1 (TNF-R_1_) (B, E, red) and interleukin-1 receptor 1 (IL-1R_1_) (H, K, red) in oligodendrocytes (arrows) in the PWM at 7 days after the hypoxic exposure and the corresponding control. The co-localized expression of APC with TNF-R_1_ and IL-1R_1_ is depicted in C and F, I and L, respectively. Note the expression of TNF-R_1_ and IL-1R_1_ is upregulated after the hypoxic exposure. Scale bars: A–L, 50 µm.

In the corpus callosum of the control rats, TNF-α as well as IL-1β expression was specifically detected in sporadic cells, confirmed to be the astrocytes by double labeling with GFAP staining (Fig. 2 A–L). At 7d following hypoxic exposure, immunoreactivity for TNF-α and IL-1β was detected and enhanced in large numbers of astrocytes (Fig. 2 D–F, J–L) when compared with the controls (Fig. 2 A–C, G–I). Expression of TNF-R_1_ and IL-1R_1_ was localized in the soma of some oligodendrocytes as confirmed by double immunofluorescence showing colocalization of APC (Fig. 3A–L). At 7d following hypoxic exposure, immunoreactivity for TNF-R_1_ and IL-1R_1_ was markedly enhanced in the soma of oligodendrocytes (Fig. 3 D–F, J–L) as compared with the control (Fig. 3 A–C_,_ G–I).

### Apoptosis of OLs in the PWM

**Figure 4:**
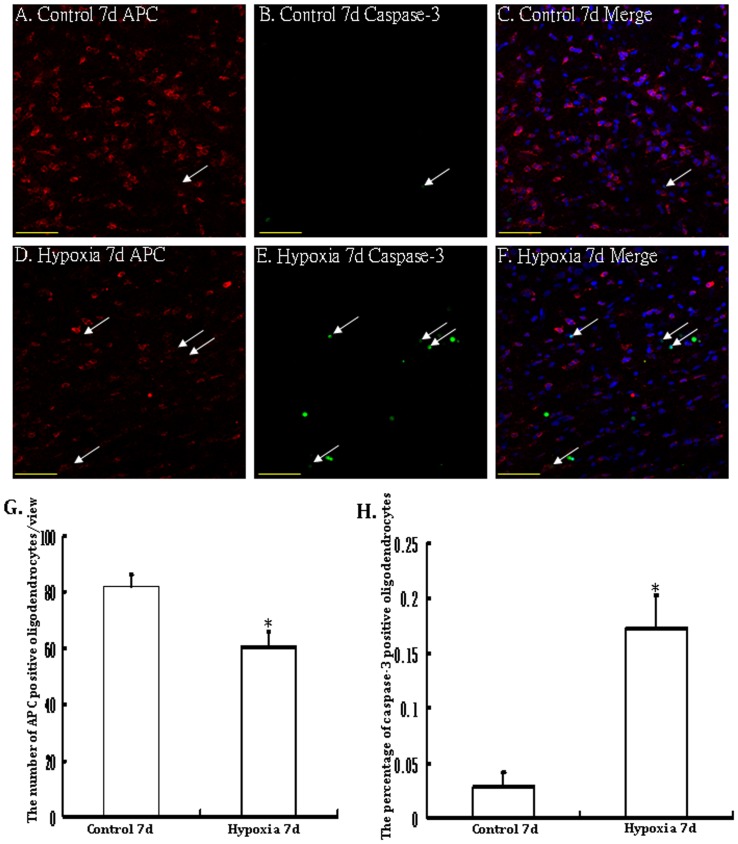
Confocal images showing APC immunolabeled oligodendrocytes (arrows)(A, D, red) and caspase-3 positive apoptotic cells (B, E; green) (arrows) as detected by double immunofluorescence in the PWM of a control and a hypoxic rat at 7d after the hypoxic exposure. The colocalized expression of APC and caspase-3 labeling can be seen in C and F. Note the increase in frequency of apoptotic nuclei in the PWM after the hypoxic exposure. Bar graphs in G and H show the significant increase in percentage of caspase-3 positive apoptotic cells (H) and decrease in the number of APC positive oligodendrocytes/view (G) in the PWM after the hypoxic exposure (* *P*<0.01). Scale bars: A-F, 50 µm.

Immunoreactive APC labeled OLs characterized by an oval cell body were identified in the PWM in the control rats (Fig. 4A–C). There was less evidence of cells marked by caspase-3 labeling (Fig. 4B). Following hypoxic exposure, however, APC immunoreactive cells showing caspase-3 labeled nucleus were observed and increased significantly (Fig. 4D–F). The percentage of caspase-3 positive oligodendrocytes at 7 days following hypoxic exposure was significantly higher than that in the corresponding controls (Fig.4 D–F, H). Another salient feature in the hypoxic rats was that the incidence of APC positive immunoreactive oligodendrocytes was decreased significantly (Fig. 4D–F, G).

### PLP Expression in the Corpus Callosum of P28d Control and P28d Hypoxic Rats

**Figure 5:**
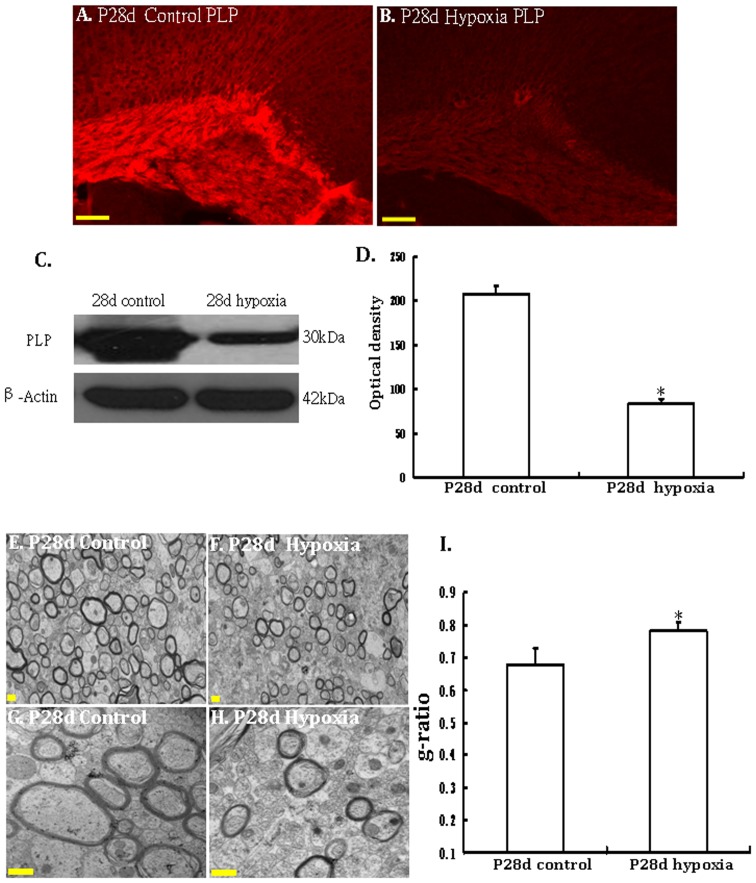
Hypoxia induces hypomyelination in the PWM in the hypoxic P28d rats. A–D show PLP protein expression in the PWM at 28 days after the hypoxic exposure and the corresponding control rats. Confocal images showing the expression of PLP in the PWM at 28 days of the hypoxic exposure (B) and the corresponding control rats (A). Note the PLP protein expression is saliently reduced following the hypoxic exposure (B) as compared with the control (A). C shows PLP (30 KDa) and β-actin (42 KDa) immunoreactive bands, respectively. Bar graph in D shows significant decrease in the optical density of PLP following hypoxic exposure when compared with the corresponding controls (*P<0.01). E-I Electron micrographs show hypomyelination and aberrant ensheathment of axons in the PWM at 28d after the hypoxic exposure. Electron microscopic images of PWM in cross sections are shown at 2 different magnifications. The number of myelinated axons is markedly decreased in the PWM at 28d after the hypoxic exposure (F) when compared with corresponding control (E). Higher magnification EM images showing thinner myelin in the PWM of hypoxic rats (G, H). I is bar graph showing increased g-ratio of myelinated axons in the PWM at 28d after the hypoxic exposure. Scale bars: A–B, 100 µm. Scale bars: E–H, 500 nm.

PLP have been very useful as markers for mature myelin sheath in CNS. When CNS myelin is decreased, PLP expression is also reduced. Immunostaining showed that PLP protein expressions were decreased in the corpus callosum of P28 hypoxic rats when compared with their age-matched littermates (Fig. 5A, B). The signal intensity of WB band was measured with Image J software. The immunoreactive bands of PLP protein levels decreased significantly in optical density in P28d hypoxic rats (Fig. 5 C–D). This finding indicates that hypoxia could lead to hypomyelination in CNS.

### Ultrastructural Study

To obtain direct evidence of hypomyelination in the CNS, we analyzed sections of the PWM in hypoxic rats and control littermates at P28 by electron microscopy. The proportion of myelinated axons was markedly reduced in hypoxic rats (Fig. 5F) when compared with the corresponding control (Fig. 5E), indicating that there was hypomyelination in the PWM of P28d hypoxic rats. We next compared myelin sheath thickness in the PWM of P28d hypoxic rats and the control by determining the g-ratio (axon diameter to total fiber diameter) of myelinated fibers. The average g-ratios were higher in hypoxic rats (Fig. 5I), indicating that myelin sheaths were thinner in hypoxic rats at P28d (Fig. 5H) compared with those of control rats (Fig. 5G). Together, these data demonstrated that there were fewer myelinated fibers in the PWM of P28d hypoxic rats, and the fibers have thinner myelin sheaths. Therefore, hypoxia induced hypomyelination in the PWM of hypoxic rats.

### *In vitro* Protein and mRNA Expression of TNF-α and IL-1β in Activated Astrocytes under Hypoxic Conditions

**Figure 6:**
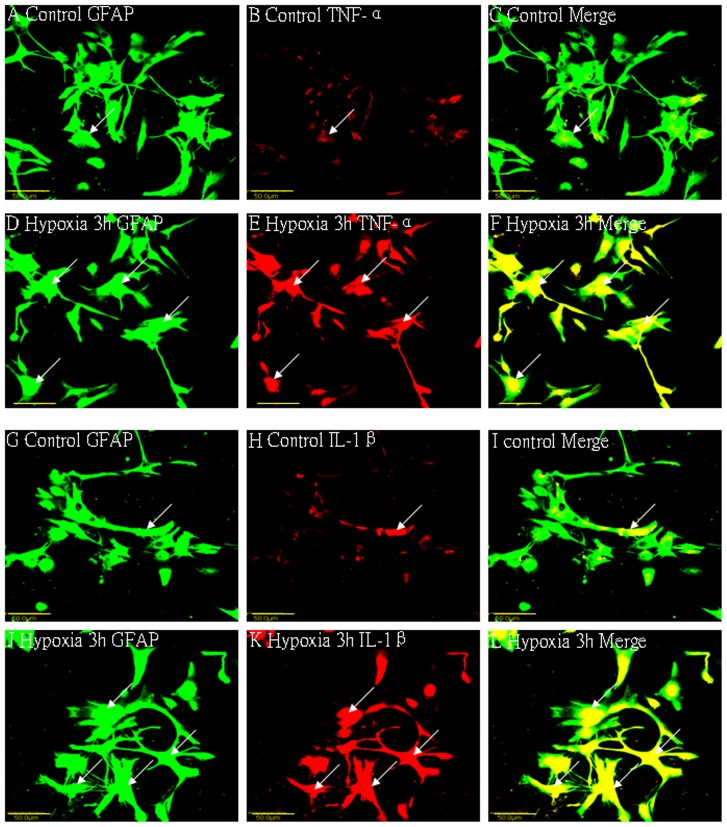
Confocal images of cultured control astrocytes showing the expression of GFAP (A, G, green), TNF-α (B red), IL-1β (H red) and co-localized expression of GFAP and TNF-α (C), GFAP and IL-1β (I). D–F show the expression of GFAP (D, green), TNF-α (E, red) and colocalized expression of GFAP and TNF-α (F) after treatment with 3% oxygen for 3 h. Note the elevated expression of TNF-α following treatment with 3% oxygen for 3 h (E) as compared with the control cells (B). J–L show the expression of GFAP (J, green), IL-1β (K, red) and colocalized expression of GFAP and IL-1β (L) after treatment with 3% oxygen for 3 h. The expression of IL-1β is greatly increased in the astrocytes after hypoxic exposure for 3 h. Scale bars: A–L, 50 µm.

TNF-α and IL-1β protein quantity increased significantly at 3 h after the hypoxic exposure as compared with the controls (p<0.05) (Fig. 1F, G). An upregulation of TNF-α and IL-1β mRNA expression was observed at 3 h after hypoxia (p<0.05) when compared with the matching controls (Fig. 1H). Double immunofluorescence labeling showed that the GFAP labeling in astrocytes was completely co-localized with TNF-α and IL-1β expression (Fig. 6A–L). At 3 h after hypoxia, the expression of TNF-α and IL-1β in astrocytes was increased significantly (Fig. 6D–F, J–L) in comparison with the control cells (Fig. 6A–C, G–I).

### *In vitro* Protein Expression of TNF-R_1_ and IL-1R_1_ in Activated Oligodendrocytes under Hypoxic Conditions

**Figure 7:**
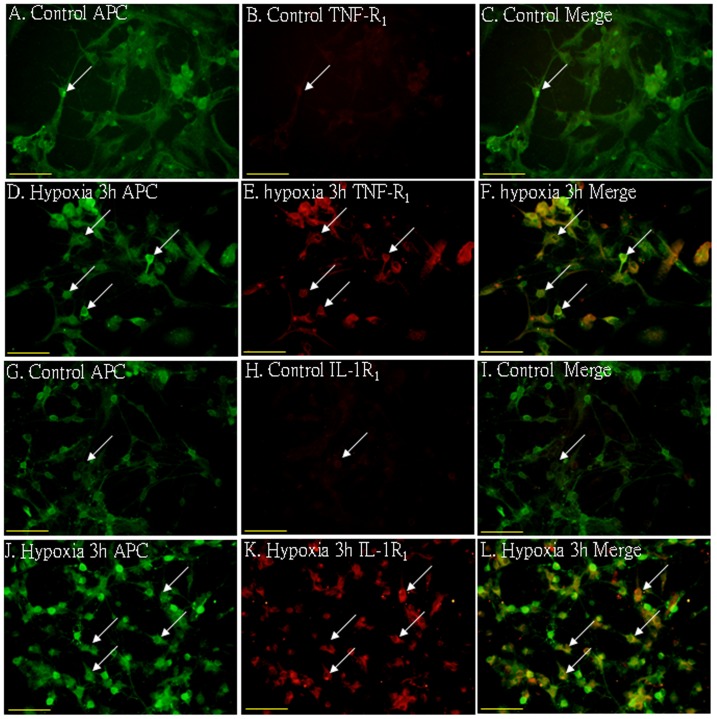
Upregulation of TNF-R_1_ and IL-1R_1_ expression in primary cultured oligodendrocytes following hypoxia. Confocal images showing TNF-R_1_ and IL-1R_1_ expression (B, E, H, K, red) in primary cultured oligodendrocytes labeled with APC (A, D, G, J, green) in both control and hypoxia for 3 h. Note TNF-R_1_ and IL-1R_1_ immunofluroscence intensity is markedly enhanced after hypoxic exposure (E, K) in comparison with the control (B, H). Scale bars: A–L, 50 µm.

Double immunofluorescence staining showed that the APC labeling in oligodendrocytes was completely co-localized with TNF-R_1_ and IL-1R_1_ expression (Fig. 7A–L). At 3 h after hypoxia, the expression of TNF-R_1_ and IL-1R_1_ in oligodendrocytes was increased significantly (Fig. 7D–F, J–L) in comparison with the control cells (Fig. 7A–C, G–I).

## Discussion

Hypoxia could result in direct and indirect brain injury. Hypoxia induced direct brain injury which occurs in the acute hypoxic phase, and indirect brain injury appears after reoxygenation which is mainly attributed to inflammatory response induced by neuro-immunological activation under hypoxic conditions [25], [26]. Neuroinflammatory response in the CNS is a complicated process which involves numerous damage signals, cellular responses and alterations in the microenvironment [27], [28]. Following the neuroinflammatory response, a complex cascade of cellular events occurs. There is now extensive evidences indicating that microglia and astrocytes, which are residential cells in the CNS, have been cited as the local immune cells and cytokine producers [29]. Microglia, which are resident immune cells in the CNS and belong to the mononuclear phagocyte system, have been recognized as sensors of brain injury and main cytokine producers [30], [31]. Other sources of proinflammatory cytokine production in the PWM may be the astrocytes. Some studies have shown that astrocytes may produce reactive responses following various kinds of injury or in neurodegenerative diseases [32]. This reactive process, known as astrogliosis or reactive gliosis, is characterized by cellular hypertrophy, hyperplasia, and an increase in immunodetectable glial fibrillary acidic protein (GFAP) [33]. Astrocytic responses may help to repair the injured CNS, but excessive astrogliosis may be detrimental and contribute to neuronal injury [34]. Reactive gliosis has the potential to overproduce free radicals and inflammatory mediators that are toxic to neurons or oligodendrocyte [34]. Since both microglial cells and astrocytes are involved in cytokine production, Sawada *et al* (34) reported the differences in roles of these cells and concluded that microglia might contribute to the early phase of cytokine production, whereas astrocytes to a late phase in brain pathologies. Microglia-derived proinflammatory mediators may promote astrocytes to generate inflammatory cytokines. This will amplify inflammatory process. Therefore, the role of microglial cells and astrocytes is synergistic in the modulation of inflammatory response in the CNS. Our previous study has shown that protein expression of TNF-α and IL-1β in the PWM was increased up to 14 days after hypoxic exposure. They were mainly detected in amoeboid microglial cells (AMC) before 7 days after hypoxic exposure. It was speculated that activated astrocytes may be the main cellular source of TNF-α and IL-1β in the protracted period of hypoxia that is, beyond 7 days.

The present study has shown an upregulation of the mRNA and protein expression of TNF-α and IL-1β in the PWM of neonatal rats at 7 days after hypoxic insult. Double immunofluorescence staining has shown that expression of TNF-α and IL-1β was detected in astrocytes as verified by their colocalization with GFAP, a cellular marker for astrocyte. This suggests that astrocytes may be the main cellular source for proinflammatory cytokine production at later time points in the PWM in hypoxic injuries. Increased cell death by apoptosis and necrosis has been found at 7 days after hypoxic exposure in the PWM of neonatal brain. The apoptotic cells were confirmed to be the APC^+^ OLs by double immunofluorescence labeling. This is coupled with upregulated expression of TNF-R_1_ and IL-1R_1_ in APC^+^ OLs at 7 days after hypoxic exposure. Therefore, we speculate that TNF-α produced by astrocytes in hypoxic conditions had induced the OL apoptosis via TNF-R_1_ on the OLs in hypoxic rats. Some studies have demonstrated that IL-1β may inhibit OL proliferation through its receptor [35]. Here, we showed increased expression of IL-1R_1_ on the APC^+^ OLs at 7 days after hypoxic exposure. Therefore, it is speculated that astrocytes-derived IL-1β in hypoxic conditions may result in PWMD via inhibition of OL proliferation.

Our *in vitro* study has shown that the mRNA and protein expression of TNF-α and IL-1β in cultured astrocytes was upregulated significantly after hypoxic exposure for 3 h. We also found that the increased expression of TNF-R_1_ and IL-1R_1_ was localized in the APC^+^ oligodendrocytes simultaneously *in vitro*. This indicates that hypoxia could activate isolated astrocytes to produce inflammatory mediators, which would damage the APC^+^ oligodendrocytes through their corresponding receptors. Therefore, astrocytes may play an important role in the pathogenesis of PWMD in the hypoxic neonatal brain.

The formation of a myelin sheath is a complex interaction between axons and OLs [36]. OLs are the myelin-forming cells in the CNS and originate from neuroepithelial cells of the ventricular zones at very early stages of embryonic life [37], [38]. OL progenitors migrate to the developing white matter tracts and undergo substantial proliferation before their final differentiation into myelin-forming cells [38]. Mature OL processes spiral around the axon to form myelin sheaths [39]. Under pathological conditions, axonal injury or OLs loss would affect a myelin sheath formation and result in hypomyelination. Our immunostaining has found reduction of APC^+^ oligodendrocytes and increased oligodendrocytic apoptosis at 7 days after hypoxic exposure in the PWM of neonatal brain. As a corollary, it is suggested that hypoxia would reduce the myelin sheath formation. PLP is the major myelin protein from the central nervous system (CNS) [40]. It plays an important role in the formation or maintenance of the multilamellar structure of myelin sheath, which function is an insulator to greatly increase the velocity of axonal impulse conduction [40]. Therefore, at present, PLP has been regarded as the marker for mature myelin sheath in CNS [40]. Under normal condition, myelin sheath formation in the PWM of rats is stable and mature at P28. Our results have shown that PLP protein expression was reduced significantly in the PWM of P28 hypoxic rats. This indicated that hypoxia has disrupted the myelin sheath formation. These findings are consistent with results by electron microscopy which has shown that there were fewer myelinated fibers in the corpus callosum of P28 hypoxic rats. The ratio of the inner axonal diameter to the total outer diameter or g-ratio is widely utilized as a functional and structural index of axonal myelination [41]. The higher g-ratio values correspond to thinner myelin sheaths [41]. Statistical analysis has demonstrated that the average g-ratios were higher in hypoxic rats, indicating that the fibers showed thinner myelin sheaths. Based on these data, we speculate that hypomyelination, a hallmark feature in PWMD, may be attributed to activation of astrocytes and production of inflammatory cytokines such as TNF-α and IL-1β, which contribute to OL damage through their corresponding receptors.

## Conclusion

This study has shown that hypoxia could activate astrocytes in the corpus callosum in the neonatal brain, which produces inflammatory cytokines such as TNF-α and IL-1β. These inflammatory mediators would induce the occurrence of apoptotic APC^+^ oligodendrocytes, thinner myelin sheath and reduction in the proportion of myelinated axons through their corresponding receptors expressed in oligodendrocytes. It is suggested that the above-mentioned inflammatory mediators generated by astrocytes under hypoxic conditions would result in hypomyelination, a hallmark in PWMD. These findings would help design a more effective therapeutic strategy in mitigating hypoxia-induced PWMD in neonatal brain by preventing the production of inflammatory mediators.
